# An overview of experimental simulations of microbial activity in early Earth

**DOI:** 10.3389/fmicb.2022.1052831

**Published:** 2023-01-12

**Authors:** Mingyu Zhao, Yao Zhao, Wei Lin, Ke-Qing Xiao

**Affiliations:** ^1^Key Laboratory of Cenozoic Geology and Environment, Institute of Geology and Geophysics, Chinese Academy of Sciences, Beijing, China; ^2^State Key Laboratory of Environmental Criteria and Risk Assessment, Chinese Research Academy of Environmental Sciences, Beijing, China; ^3^Key Laboratory of Earth and Planetary Physics, Institute of Geology and Geophysics, Chinese Academy of Sciences, Beijing, China; ^4^School of Earth and Environment, University of Leeds, Leeds, United Kingdom

**Keywords:** early Earth, microbes, biogeochemical cycles, experimental simulations, interactions

## Abstract

Microbial activity has shaped the evolution of the ocean and atmosphere throughout the Earth history. Thus, experimental simulations of microbial metabolism under the environment conditions of the early Earth can provide vital information regarding biogeochemical cycles and the interaction and coevolution between life and environment, with important implications for extraterrestrial exploration. In this review, we discuss the current scope and knowledge of experimental simulations of microbial activity in environments representative of those of early Earth, with perspectives on future studies. Inclusive experimental simulations involving multiple species, and cultivation experiments with more constraints on environmental conditions similar to early Earth would significantly advance our understanding of the biogeochemical cycles of the geological past.

## Introduction

**Figure 1:**
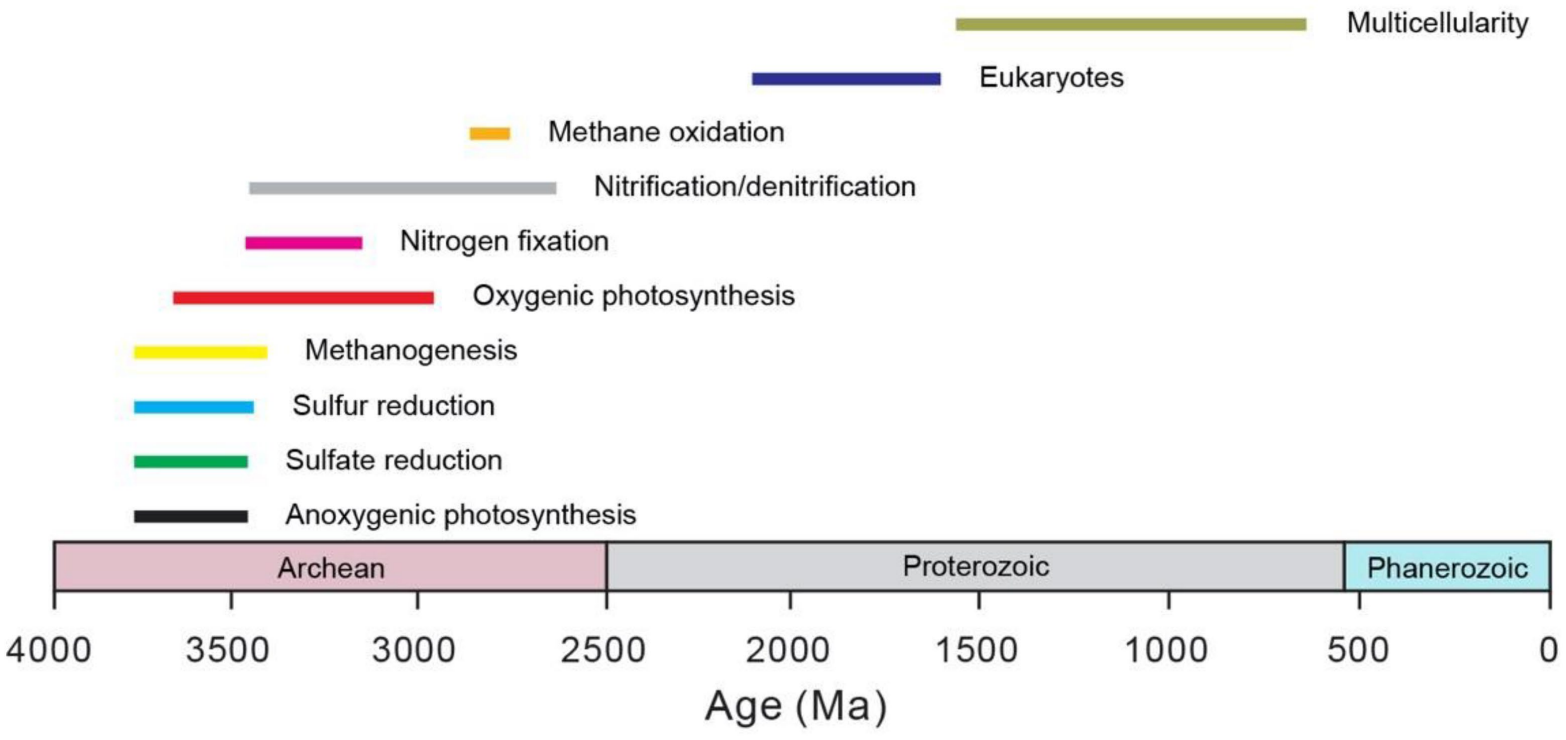
Origin of major microbial metabolisms and groups through time. The bars represent the uncertainty in the estimation. The data are mainly from Moore et al. (2017). Note that the microbes before the rise of eukaryotes all belong to prokaryotes. The age for the origin of eukaryotes comes from Knoll et al. (2006). The age for the origin of multicellular eukaryotes comes from Zhu et al. (2016) and the references therein.

Microbes are the main drivers for global biogeochemical cycles throughout the Earth history (e.g., Falkowski et al., 2008). In fact, microbial activity not only sustains the habitable environments of the Earth surface, but also is responsible for the gradual development of an oxygen-rich atmosphere that paved the way for the rise of eukaryotes (e.g., Lyons et al., 2014). Life only existed as single-cell microbes before the appearance of multicellular eukaryotes, perhaps as early as the Mesoproterozoic (e.g., Zhu et al., 2016; Figure 1). Thus, these primitive microbes were important in shaping the biogeochemical cycles as well as the environmental conditions of early Earth.

**Figure 2:**
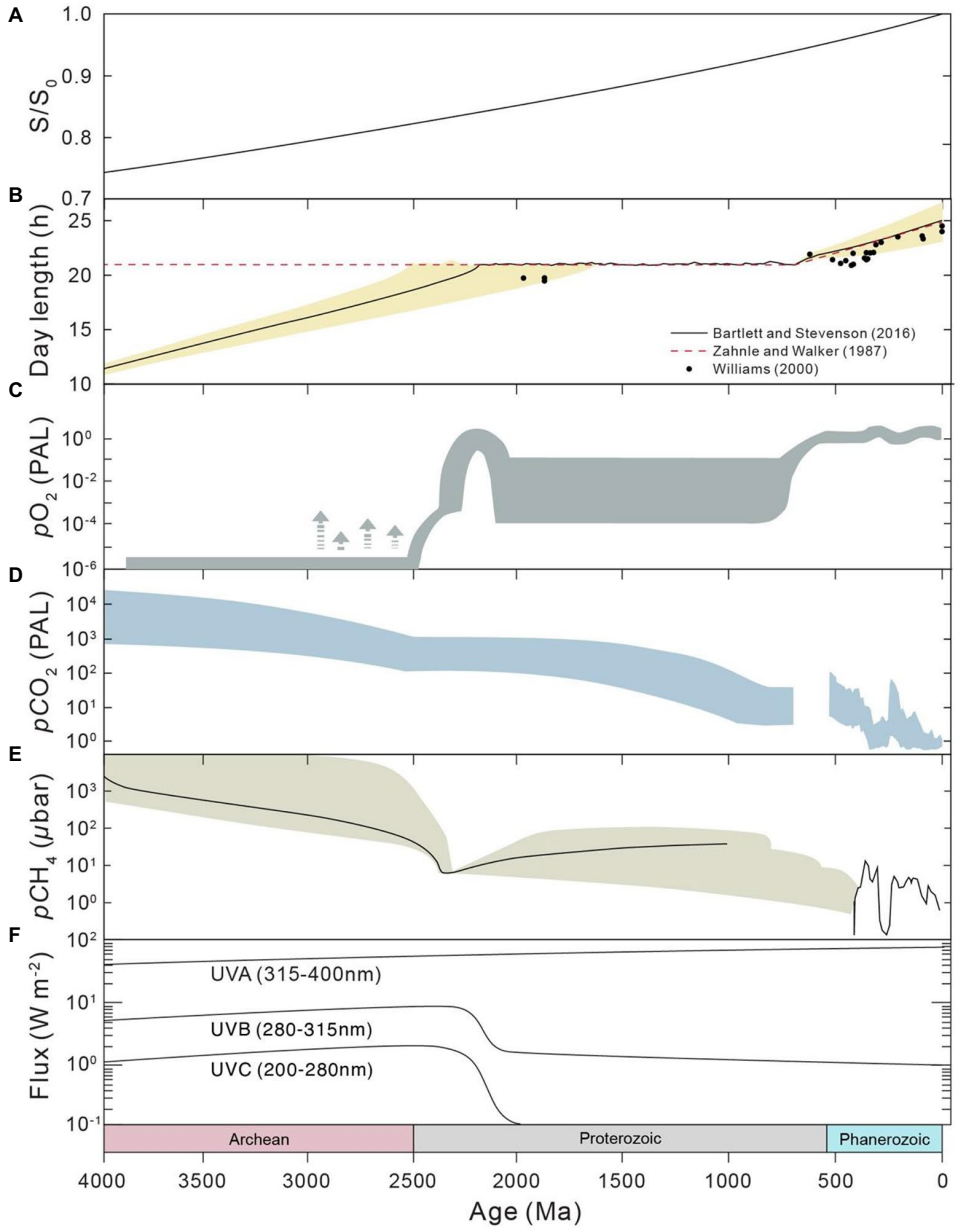
Evolution of astronomical conditions and atmospheric composition throughout Earth history. **(A)** Solar luminosity, derived from Kasting (1987). **(B)** Day length. **(C)** Atmospheric *p*O_2_, after Lyons et al. (2014). **(D)** Atmospheric *p*CO_2_. Results of the Precambrian (left) are from Kasting (1987), whereas those of the Phanerozoic (right) are from Royer et al. (2004). **(E)** Atmospheric *p*CH_4_, derived from Catling and Zahnle (2020). **(F)** Ultraviolet (UV) radiation at the ground, modified from Cockell (2000).

**Figure 3:**
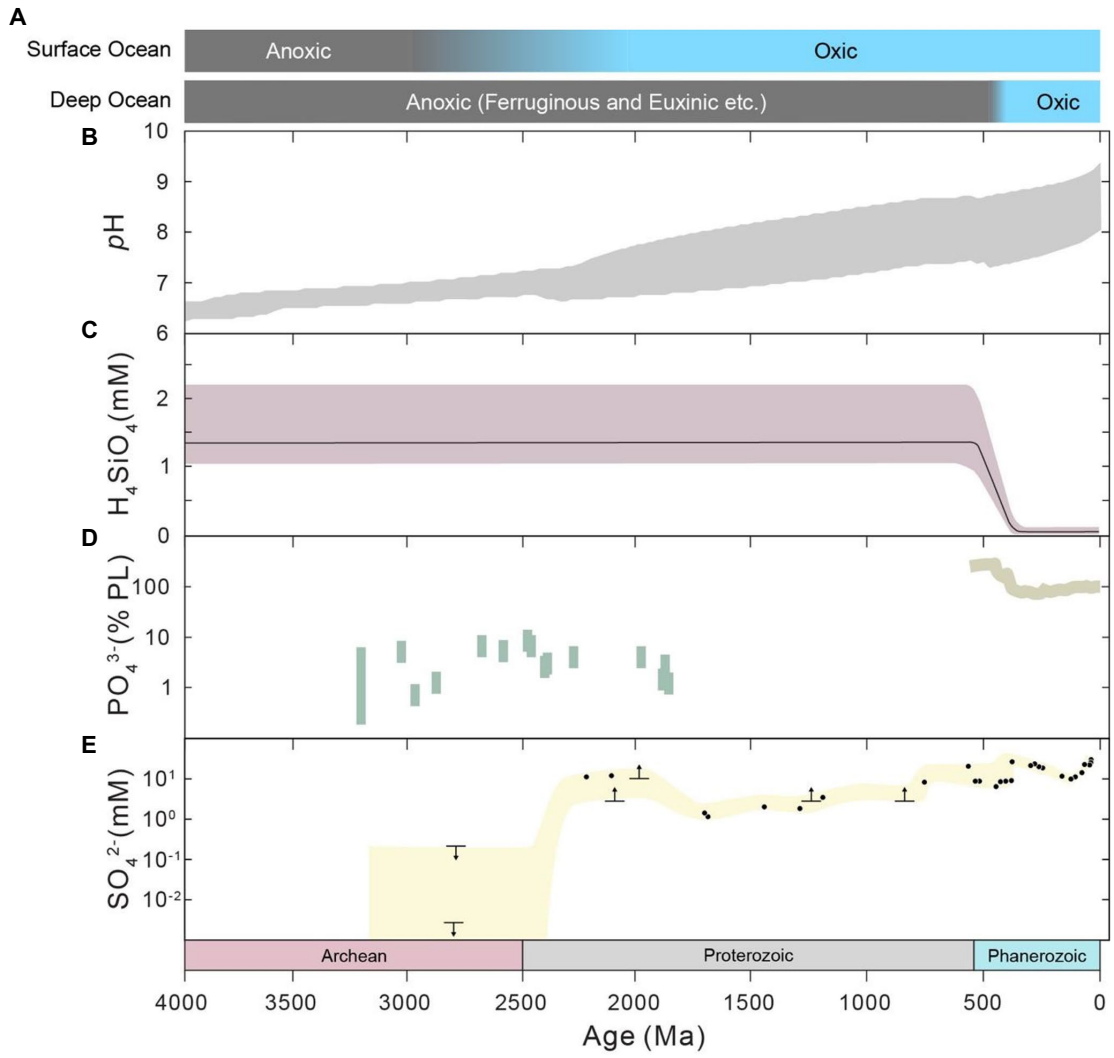
Evolution of seawater conditions and composition through Earth history. **(A)** Redox state, after Sperling et al. (2015). **(B)** Marine pH, which is the results within the 95% confidence interval from Halevy and Bachan (2017). **(C)** Marine silica concentration, from Isson and Planavsky (2018). **(D)** Marine dissolved phosphate concentration. Results of the Precambrian (left) are from Bjerrum and Canfield (2002), Konhauser et al. (2007) and Jones et al. (2015), which were constrained by the Fe/P ratios of iron-rich sediments. The results of the Phanerozoic (right) are from the model results of Lenton et al. (2018). **(E)** Marine sulfate concentration, taken from Habicht et al. (2002), Planavsky et al. (2012) and references therein, Crowe et al. (2014) and Blättler et al. (2018).

The environments of Earth surface during the Precambrian (before the Phanerozoic Eon) could be fundamentally different from those of modern times and the Phanerozoic. Firstly, the astronomical conditions of the Precambrian were significantly different from modern conditions. The solar luminosity has steadily increased throughout the history of the Earth, from ~70% of the present value in the Precambrian (e.g., Gough, 1981; Kasting, 1987), according to standard solar evolution theory. The daylength has also increased during geological history due to the change in Earth’s rotation rate (e.g., Munk and MacDonald, 1960; Zahnle and Walker, 1987; Williams, 2000; Bartlett and Stevenson, 2016; Figures 2A,B). It is generally accepted that daylength increases with time as the rotation rate of the Earth decreases due to tidal friction (e.g., Munk and MacDonald, 1960). The daylength may have had a long period of stasis at 21 h from ~2,200 to 600 Ma and potentially in prior periods, owing to the resonance between Earth’s rotation and the semidiurnal atmospheric thermal tide (Zahnle and Walker, 1987; Bartlett and Stevenson, 2016). In addtion, the oxygen level of the Precambrian atmosphere could have been much lower than the modern level (e.g., Farquhar et al., 2000; Lyons et al., 2014), whereas the *p*CO_2_ and *p*CH_4_ levels of the Precambrian could have been much higher (e.g., Kasting, 1987; Zhao et al., 2018; Catling and Zahnle, 2020; Figures 2C,D). The atmospheric oxygen level was likely lower than 0.001% of the present atmospheric level during the Archean period (e.g., Pavlov and Kasting, 2002; Lyons et al., 2014). The atmospheric oxygen level during the Proterozoic is highly debated, with estimates in the range of <0.1 to 40% (Canfield, 1998; Rye and Holland, 1998; Planavsky et al., 2014, 2020; Canfield et al., 2021). Furthermore, the chemical composition, redox status and *p*H of ocean water (Figure 3) during the Precambrian could have been significantly different from those of today. Ferruginous conditions may have been a predominant state for the Precambrian ocean (e.g., Sperling et al., 2015), while the modern ocean is largely oxic. Seawater calcium, bicarbonate, silica and barium concentrations could have been much higher (Walker, 1977; Halevy and Bachan, 2017; Isson and Planavsky, 2018), but sulfate and carbonate ion concentrations could have been much lower during early Earth (Walker, 1977; Habicht et al., 2002). The concentrations of trace components have also varied with time, mainly due to the change in seawater redox conditions (Moore et al., 2017). Marine pH in early Earth was expected to be much lower than what is found today (Halevy and Bachan, 2017; Isson and Planavsky, 2018), largely because of the high atmospheric *p*CO_2_ during this period. Finally, the flux of ultraviolet (UV) radiation to the Earth surface before the Great Oxidation Event (GOE) at the end of Archean could have been orders of magnitude higher than that of today (e.g., Cockell, 1998; Rettberg et al., 1998), which is due to the rise in atmospheric oxygen and the corresponding production of ozone during the GOE.

As a perfect modern analogue for Precambrian environments is lacking, experimental simulation of microbial activity under artificial Precambrian conditions is an instructive method of investigating the adaptive strategies of microorganisms under the extreme environmental conditions of early Earth (e.g., high UV radiation) as well as the mechanisms, kinetics and evolution of biogeochemical cycles. Moreover, microbial biomineralisation could be one of the main drivers for the formation of unique deposits in the Precambrian, such as banded iron formations (BIFs) and substantial stromatolites. Thus, biomineralisation experiments can provide vital information regarding the mechanism and conditions for the formation of Precambrian chemical sediments. Here, we review recent advances in microbial experiments on these topics, although we acknowledge that well-calibrated ecosystem simulation models, based on predicted physicochemical parameters and expected biogeochemical activities, could also be helpful in simulating Earth’s early environments (e.g., Herman and Kump, 2005; Ozaki et al., 2018; Zhao et al., 2018). There are also extensive studies on microbial activities in hydrothermal vents, which have important implications on the origin of life and the early environments of the Earth. These have been reviewed elsewhere (e.g., Martin et al., 2008; Xiao and Yu, 2014; Weiss et al., 2016).

## Ultraviolet radiation

High levels of UV radiation exist not only in the space environment but also in the early Earth. Thus, studies on the response of microbes upon exposure to UV radiation are important in elucidating the selective pressure and ecology niche of microbes on early Earth (Sagan, 1973; Cockell et al., 2011). In addition, these types of experiments are also important for understanding the potential of the natural transport and spread of microbes between planets and finding microbes that are suitable for food and oxygen production or geoengineering in space exploration (e.g., Olsson-Francis and Cockell, 2010a,b; Cockell et al., 2011). In this section, we focus on the application of UV radiation experiments that have implications for learning about the early Earth biosphere. In the Archean period, microbes resident on the Earth surface might have been exposed to UV influx with wavelengths >200 nm, whereas the influx of UV radiation on Earth today is limited to wavelengths >290 nm (Figure 2F, e.g., Rettberg et al., 1998; Cockell, 2000). However, some suggest that the early atmosphere might have been a UV screen (e.g., Sagan, 1973; Kasting, 1993). UV >200 nm radiation could generate more than three orders of magnitude greater damage to DNA (e.g., Rettberg et al., 1998). Such radiation could also cause strong damage to proteins, lipids and pigments (e.g., Tevini, 1993; Cockell, 1998). Although UV radiation is a strong selective pressure for microbes, the presence of physical protection, biological protection, biological adaption and repair ability (Cockell, 1998) might have made UV radiation not a critical limitation to microbes in early Earth (e.g., Pierson et al., 1993). Extensive cultivation experiments under artificial UV conditions of the early Earth have been performed to understand the mechanism for the survival of microbials under high UV radiation, which will be discussed in the following sections.

### Physical protection

Various screening methods under high UV radiation have been proposed and experimentally studied (Cockell, 1998), including physical protection and biological protection. A variety of substances were found to have the ability of physical protection, such as water, iron compounds (Pierson et al., 1993; Bishop et al., 2006; Gómez et al., 2007; Gauger et al., 2016), Fe(III)-silica precipitates (Phoenix et al., 2001; Mloszewska et al., 2018), clays (Kugler and Dong, 2019), minerals (Garcia-Pichel and Belnap, 1996), porous rocks (Cockell et al., 2002; Bryce et al., 2015), nitrogenous salts (Margulis et al., 1976), π-electron-containing chromophores (Sagan, 1973; Cockell, 1998), organic compounds (Cleaves and Miller, 1998), silica (Phoenix et al., 2001, 2006) and dead cells (Margulis et al., 1976). Water bodies, such as lakes, rivers and oceans, could also act as an effective shield through the attenuation effect (Sagan, 1973; Margulis et al., 1976). As Fe(II) and silica were both enriched in Archean ocean water (Maliva et al., 2005; Czaja et al., 2012), understanding the screen effect of Fe(III)-silica precipitates on UV radiation can provide important insight into the microbial ecosystems in the Archean ocean (Mloszewska et al., 2018). The transmission of UV radiation through gels of FeCl_3_ was measured by Pierson et al. (1993). They found that a 1 mm gel of 0.1% FeCl_3_ can reduce UV radiation to 1% of its initial intensity. Meanwhile, with respect to radiation used in photosynthesis, 85% of visible radiation and 93% of near infrared (NIR) radiation can pass through the same gels. Such an experiment demonstrates that iron compounds at the surface of mats or dispersed iron compounds in water bodies can protect the underlying microbials from UV radiation. Meanwhile, nanograins of iron (hydro)oxides formed by certain anoxygenic phototrophic Fe(II) oxidisers such as *Rhodopseudomonas palustris* strain TIE-1 and Rhodobacter ferrooxidans strain SW2 can be loosely attached to the cell surfaces and protect the cells from UV radiation (Gauger et al., 2016). Cultivation of cyanobacteria from hot springs in artificial Archean seawater (iron-silica solution) revealed that the formation of iron-enriched silica gels and crusts could efficiently protect the bacteria against UV radiation (Phoenix et al., 2001). Mloszewska et al. (2018) further investigated the protection efficiency of Fe(III)-silica precipitates in Archean ocean water against UV radiation. They found that both Fe(III) and silica can efficiently screen UV radiation. UV-C is attenuated by 18% in Si-only solution, 32% in Fe(III)-only solution and 56–70% in Fe(III)-silica solution. Furthermore, they have used a model to show that UV-C is attenuated by ~80% in Archean seawater analogues with 100 nM Fe(II). However, although their cultivation experiment on planktonic cyanobacteria *Synechococcus* reveals much higher survival rates in Fe(II)-silica media (3%) than in un-supplemented media (0.13%), the high mortality rate (97%) even under the protection of Fe(III)-silica precipitates suggests that the ecology niche of plankton and its primary productivity in the Archean ocean still might have been strongly limited by the UV radiation. Kugler and Dong (2019) examined the protection efficiency of three mica minerals (muscovite, phlogopite and biotite) against UV radiation. They found that biotite has the best ability to screen UV radiation, owing to the Fe(II) content in biotite, which is the highest among the three minerals.

Cyanobacteria mats are likely the most important ecosystem on land during the Archean period (e.g., Planavsky et al., 2021). In this case, due to the shielding effect of water and minerals, the surface of mats can effectively attenuate UV radiation and protect microorganisms from damage from UV fluxes (Garcia-Pichel and Belnap, 1996). Margulis et al. (1976) observed that cells in the interior of a mat can survive after 3 days of continuous UV irradiation, even if the cells on the surface died after minutes. This suggests that the cells in the interior were excellently protected by the surface cells. Nitrogenous salts in solution can also protect microbials due to their strong adsorption ability on UV radiation (Margulis et al., 1976). Cleaves and Miller (1998) have proposed that prebiotic organic polymers such as cyanide polymer and spark discharge polymer in seawater might have acted as efficient UV adsorbers that can protect probiotic organic compounds. In an experiment outside of the International Space Station, it was found that *Chroococcidiopsis* could survive after exposure to UV radiation in space (>110 nm or 200 nm) for 548 days, although other microbes cannot recover from such detrimental exposure (Cockell et al., 2011). Self-shielding due to multiple layers of cells could be an important factor in maintaining the survival of *Chroococcidiopsis* (Cockell et al., 2011). This study suggests that, even under the worst-case scenario of UV exposure, some microorganisms might have had the potential to occupy the land during the Archean period without any protection by physical screening.

### Biological protection

Experimental studies on modern microbes have also revealed several biological mechanisms that serve to screen UV radiation, including negative UV phototaxis (Bebout and Garcia-Pichel, 1995), compression of spirals (Wu et al., 2005) as well as the production of pigments and organics for UV screening (Garcia-Pichel et al., 1992, 1993; Ehling-Schulz et al., 1997; Oren, 1997; Sinha and Häder, 2002, Sinha and Häder, 2008). A vertical migration strategy for cyanobacteria was found in both mats and water columns (e.g., Reynolds et al., 1987; Garcia-Pichel et al., 1994). Bebout and Garcia-Pichel (1995) have found that photoautotrophic microbes in the cyanobacteria mat can migrate downward upon the application of UV radiation, which results in a color change in the surface of mats as well as downward displacement of the maximum oxygen layer. Experiments by Garcia-Pichel et al. (1992, 1993) show that extracellular pigment scytonemin from terrestrial cyanobacterium *Chlorogloeopsis* sp. can reduce UV radiation (365 nm) by 70%, whereas mycosporine-like amino acids of terrestrial cyanobacterium *Gloeocapsa* sp. can reduce UV radiation (320 nm) by ~20–30%. Moreover, a variety of biological repair processes, such as photolyase photoreactivation, DNA excision repair, SOS response and post-replication repair, have also been found and well-investigated (e.g., Sutherland, 1981; O’Brian and Houghton, 1982; Owttrim and Coleman, 1989; Friso et al., 1994; Sancar, 1994). Some magnetotactic bacteria can produce intracellular iron nanoparticles, which could have been used for mitigating the stress from UV and free-iron-generated reactive oxygen species (ROS) in early Earth (Lin et al., 2019; Liu et al., 2022). Microbial adaption to high-UV radiation through mutation might have occurred during the early Earth. Studies have showed that microbial populations can evolve under continued UV stress and gradually develop the resistance to UV radiation (Wassmann et al., 2010). For example, Luckiesh and Knowles (1948) revealed that *Escherichia coli* can double their resistance to UV radiation through five times repeated exposure to UV radiation. In a space experiment, Wassmann et al. (2010) found that populations of *Bacillus subtilis* can increase their UV resistance by a factor of three after approximately 700 generations of periodic UV radiation. It should be noted that ancient microorganisms might have possessed additional unknown protection mechanisms during the early Earth that have now been lost due to evolutionary redundancy under the current exposure conditions. As these could be difficult to re-acquire under short-term evolutionary selection, we may not be able to observe their full potential through experiments on present-day microorganisms.

## Biogeochemical cycles

### Microbial mats

Microbial mats might have been an important player in global biogeochemical cycles during the early Earth. Microbial mats are microbial ecosystems that consist of vertically distributed layers of pigmented bacteria that can harvest energy from the chemical gradients at the boundary between soils/sediments and water (e.g., Ludwig, 2004). In modern environments, cyanobacteria-dominated mats are restricted in various harsh settings, including hypersaline salterns and deserts (e.g., Bebout et al., 2004; Rodriguez-Caballero et al., 2018). However, before the occurrence of plants and bioturbation during the Phanerozoic, the land and perhaps marginal marine were likely covered mainly by microbial mats (e.g., Dornbos, 2006; Lalonde and Konhauser, 2015), which might have acted as a major source and/or sink for some global biogeochemical components such as CH_4_ and O_2_. Petrographic and geochemical evidence suggests the occurrence of microbial mats on river systems as early as 3.22 Ga (Homann et al., 2018).

In a landmark study, Hoehler et al. (2001) revealed that modern cyanobacteria mats can release reducing gases such as H_2_, CO and CH_4_, which would have been important for the biogeochemical cycles during the early Earth. For example, the generation of H_2_ might have resulted in an H_2_-rich atmosphere and the escape of H_2_ to space, which might have been the mechanism for the gradual oxygenation of the atmosphere (Walker, 1977; Hoehler et al., 2001). Moreover, the release of CH_4_ might have warmed the climate of the early Earth, a hypothesis that has received much attention in the literature (e.g., Bebout et al., 2004; Kelley et al., 2006; Zhao et al., 2018).

Both cultivation experiments and biogeochemical models have been used to clarify the potential role of microbial mats in the climate of the Precambrian (Bebout et al., 2004; Kelley et al., 2006; Zhao et al., 2018). During the majority of the Precambrian, the Earth was in a clement climate, although the solar luminosity was lower than that in modern times (e.g., Gough, 1981). Microbial mats might have been a solution to this “faint young sun” paradox, as it could release substantial CH_4_ under the low atmospheric oxygen level of the Precambrian, which would have warmed Earth’s climate. Cultivation experiments were designed to understand the change in methane release in the coastal marine mats under low SO_4_^2−^ conditions during the Archean period (Bebout et al., 2004; Kelley et al., 2006). At low SO_4_^2−^ (<0.2 mM), methane fluxes increased by 10-fold as sulfate reduction was out-competed by methanogenesis during organic matter remineralisation, but the remineralisation by methanogenesis was still insignificant (0.4%) relative to the total carbon release by mats. However, as has been mentioned in Bebout et al. (2004), the rates of net methane production still showed a linear increase at the end of the experiments, which represents an important caveat that the experiment might have not reached steady state. In another experiment with low SO_4_^2−^ concentration (<1 mM), methane production was found to reach as high as 7% of the total carbon degradation (Kelley et al., 2006). Therefore, there is still a large uncertainty in the estimate of the potential contribution of CH_4_ from costal marine mats to the climate of early Earth. If 7% of the Archean primary production (~800 Tmol/yr. according to Lalonde and Konhauser, 2015) was transferred to CH_4_ in the atmosphere, this would result in a methane flux of ~56 Tmol/yr, which would likely have a large influence on the climate of early Earth (e.g., Zhao et al., 2018). It is worthy to note that there is a large uncertainty in the estimate of the primary productivity of the Archean. While UV radiation may have been harmful to the productivity of early mats, high atmospheric *p*CO_2_ could have been a factor that could significantly elevate the productivity (e.g., Ji et al., 2020).

However, it must be noticed that the Archean period is not only characterised by low seawater SO_4_^2−^ but also low atmospheric oxygen. Oxygen in the ocean and atmosphere should have had a big influence on the methane flux of both terrestrial and marine microbial mats at night, due to the existence of aerobic methane oxidation in the mats. Thus, the presence of oxygen might have been a reason for the relatively low methanogenesis rate in the experiment of Bebout et al. (2004). Through a biogeochemical model, it was demonstrated that terrestrial microbial mats could release substantial CH_4_ under low atmospheric oxygen during early Earth (Zhao et al., 2018). Considering that the SO_4_^2−^ concentrations in terrestrial water bodies such as rivers, lakes and wetlands should be even lower than that of seawater (Zhao et al., 2018), the potential of CH_4_ release by terrestrial mats can be higher.

As microbial mats were one of the major ecosystems in early Earth, they have also shaped the long-term evolution of other global biogeochemical cycles, such as those of nitrogen and oxygen. Thomazo et al. (2018) argued that microbial mats such as biological soil crusts (BSCs) could have been an important source of N components such as ammonium and nitrate, which might have played a significant role in the early evolution of the global nitrogen cycle. As revealed by a recent study by Klatt et al. (2021), the net productivity and thus oxygen generation in cyanobacterial mats are positively correlated with the daylength. The authors further proposed that the increase in daylength and thus oxygen generation from microbial mats with time could be one of the reasons for the stepwise oxygenation of the Earth surface. Microbial mats could also have some indirect effect on global biogeochemical cycles. For example, oxygen produced by mats could have generated oxygen oases at the top of the mats, which could result in the oxidative weathering of sulfur and redox-sensitive trace metals before the rise of atmospheric oxygen (Lalonde and Konhauser, 2015). The oxidative weathering related to microbial mats could be the reason for pre-GOE signals of oxidation (Lalonde and Konhauser, 2015). Note that the oxygen oases at the top of the mats only occurred at daytime during the Precambrian due to photosynthesis, and it would have disappeared during night as the oxygen level of the atmosphere was reduced (e.g., Zhao et al., 2018). This would have resulted in a high CH_4_ flux from the mats to the atmosphere during the night.

### Environmental forcings for primary productivity

Primary production is one of the main drivers for global biogeochemical cycles. It is therefore important to understand the forcings for primary productivity in early Earth. Several experimental studies have been conducted to investigate the response of primary productivity to Precambrian conditions, such as high seawater Fe(II), high atmospheric *p*CO_2_, and unique ecophysiological mechanisms such as competition for nutrients between oxygenic cyanobacteria and Fe(II)-oxidising anoxygenic photosynthesizers (Swanner et al., 2015; Kamennaya et al., 2018; Ozaki et al., 2019; Herrmann et al., 2021; Szeinbaum et al., 2021). Although extant organisms used in the studies described almost certainly cannot be mapped to those present in ecosystems on the early Earth, we can reasonably assume that their core metabolic pathways and ecological activities had equivalents in those early ecosystems (e.g., Falkowski et al., 2008).

As ferruginous seawater conditions were probably widespread in the Precambrian (e.g., Sperling et al., 2015), it is important to understand the influence of Fe(II)-rich seawater on oxygenic photosynthesis, which elucidates the history of oceanic and atmospheric oxygenation. Through cultivation experiments, Swanner et al. (2015) found that both the growth rates and efficiency of oxygenic photosynthesis of planktonic cyanobacterium *Synechococcus* PCC 7002 decrease under high Fe(II) concentrations (>50 μM). The authors further proposed that Fe(II) toxicity of cyanobacteria under the conditions of Fe(II) upwelling might have decreased the oxygen generation rate in the photo zone in the oceans of early Earth, determining the onset of the GOE.

However, several lines of evidence show that Fe(II) toxicity might have not significantly limited the expansion of cyanobacteria in the early oceans (Ward et al., 2019; Szeinbaum et al., 2021). Firstly, many types of terrestrial cyanobacteria can survive at high Fe(II) concentrations (10–150 μM), which characterised the Archean oceans (Brown et al., 2005; Thompson et al., 2019; Ward et al., 2019). Next, microbial “helpers” such as facultative anaerobic gammaproteobacterial *Shewanella* using an ROS defence strategy, might have partially relieved the toxicity of Fe(II) on cyanobacteria (Szeinbaum et al., 2021). This is because the Fe(II) toxicity of cyanobacteria could be generated by the hydroxyl radicals from the reaction between Fe(II) and ROS formed during photosynthesis (Swanner et al., 2015; Szeinbaum et al., 2021). Finally, experimental setup and strain selection may have a great influence on the conclusion (Herrmann et al., 2021). Previous experiments examining Fe(II) toxicity were performed in a closed system (Swanner et al., 2015). In the cultivation experiments on cyanobacteria *Pseudanabaena* sp. *PCC7367* and *Synechococcus* sp. *PCC7336*, Herrmann et al. (2021) analysed the influence of experimental setup (open vs. closed system) on the observation of Fe(II) toxicity. In their experiments, Fe(II) toxicity was not observed in open-system cultures with continuous gaseous exchange. They further suggested that closed systems are not suitable for simulating the Archean environments, as the varying *p*CO_2_ concentration during the experiment could have a significant impact on the experimental results.

Other than Fe(II) toxicity, the competition of nutrients such as P between oxygenic cyanobacteria and Fe(II)-oxidising anoxygenic photosynthesizers (photoferrotrophs) might also have had an influence on productivity and oxygen release in the early oceans (Ozaki et al., 2019). These photoferrotrophs inhabit at a deeper water depth than oxygenic cyanobacteria, which are located closer to the source of nutrients in the deep ocean water. Photoferrotrophs also undergo high-affinity PO_4_^3−^ metabolism. These competitive advantages possessed by photoferrotrophs might have decreased the productivity and oxygen release of oxygenic cyanobacteria, resulting in the delayed oxygenation of the ocean and atmosphere system.

### Other studies on biogeochemical cycles

Besides studies on microbial mats and primary productivity, other aspects of global biogeochemical cycles of early Earth have been experimentally investigated, such as the nitrogen cycle (Michiels et al., 2017), biological pumps (Kamennaya et al., 2018) and chemical weathering (Zaharescu et al., 2019). Through an incubation experiment in a modern ferruginous basin, an analogue for the Precambrian ocean, Michiels et al. (2017) reveals that a large fraction (40%) of NO_3_^−^ is reduced to NH_4_^+^ rather than lost to the atmosphere as N_2_. The transformation of NO_3_^−^ to NH_4_^+^ would promote the retention of N in seawater, further boosting primary productivity. Using a biogeochemical model, Michiels et al. (2017) further suggested that the global primary productivity in the ferruginous ocean might have been limited by P rather than N. The biological pump is an important process in global C and O cycles, as it influences the final retention of C in marine sediments, further impacting the accumulation of O_2_ in the atmosphere. It was suggested that the efficiency of the biological pump was low during the early Earth due to the lack of a ballast with a high sinking rate from a predator (Logan et al., 1995). However, it was recently found that the conditions of high *p*CO_2_ in the early Earth can promote the formation of acidic extracellular polysaccharides (EPS; Kamennaya et al., 2018). These EPS can aggregate the dead cells of planktonic cyanobacteria to a ballast, increasing the efficiency of the biological pump.

## Mineral formation

### Banded iron formation

The sediments of the Precambrian could be different from those found in today’s environments. In particular, a substantial number of BIFs with alternating layers of iron minerals and silica were formed in the Precambrian (e.g., Konhauser et al., 2017), in contrast to the lack of such deposits during the Phanerozoic. Both abiotic and biotic models of BIF formation have been proposed and intensely investigated. Many microbial experiments have been performed to investigate the mechanism of BIF formation, which has been reviewed in Posth et al. (2013, 2014) and will only be briefly discussed here.

Two biological processes may have contributed to the original precipitation of iron minerals of BIF. Firstly, Fe(II) can be oxidated to ferric hydroxide by O_2_ generated by planktonic bacteria such as cyanobacteria. In this case, ferric hydroxide is the indirect product of the microbial process. Secondly, photoferrotrophy that use Fe(II) as an electron donor can produce Fe(III) (Garrels et al., 1973). The anoxygenic photoautotrophic bacteria used in photoferrotrophy could live below the layer of cyanobacteria. The anoxygenic bacteria thus has a competitive advantage to oxidate Fe(II), as it is closer to the source of Fe(II) in the Precambrian ocean, which is thought to be hydrothermal (e.g., Holland, 1973). Calculations based on the results of microbial experiments suggest that the anoxygenic photoautotrophic bacteria could account for the formation of most, if not all, of the initial iron minerals in BIF (Kappler et al., 2005). Furthermore, microbial processes such as microbial Fe(III) reduction could also contribute to the formation of minerals with Fe(II) such as magnetite and siderite in BIF (e.g., Konhauser et al., 2005; Li et al., 2011; Köehler et al., 2013; Halama et al., 2016; Bauer et al., 2020).

The formation of BIF through photoferrotrophy also requires the separation of biomass from Fe(III) minerals. Their co-precipitation could result in intense respiration in sediment, which could preclude the preservation of Fe(III) in BIF. However, data indicate the richness of Fe(III) in BIF, with an average Fe oxidation state of 2.4–2.6 (Posth et al., 2013; Thompson et al., 2019). A recent experiment suggests that the cell surface of a photoferrotroph could repel iron oxides under silica-rich seawater (Thompson et al., 2019), which can explain the lack of organic matter and the preservation of Fe(III) in BIF. Microbial cultivation experiments were also conducted to more precisely simulate the rate of photoferrotrophy under early Earth conditions. Konhauser et al. (2007) simulated the biological and abiotic oxidation of Fe(II) under disequilibrium water chemistry when Fe(II)-rich hydrothermal fluids mixed with Precambrian seawater that was rich in silica and HCO_3_^−^. They found that abiotic oxidation of Fe(II) was not quick enough to compete with the precipitation of Fe(II) silicates, whereas photoferrotrophy could induce rapid formation of Fe(III) hydroxide. The presence of silica could increase the oxidation rate of Fe(II) by green-sulfur bacteria *Chlorobium ferrooxidans* KoFox (Gauger et al., 2016). On the other hand, Croal et al. (2009) found that the rate of phototrophic Fe(II) oxidation by purple bacteria *Rhodopseudomonas palustris* and *Rhodobacter* species would not have been significantly influenced by high atmospheric H_2_ under the high bicarbonate concentrations of the Archean.

The mechanisms for the formation of alternative bands of iron- and silica-rich minerals in BIF have also been studied through laboratory experiments (Posth et al., 2008; Schad et al., 2019). One possibility is that the iron- and silica-rich bands were formed under different temperatures. Whereas the rate of Fe(III) minerals formed by anoxygenic photoautotrophic bacteria is highest between 20 and 25°C, abiotic silica precipitation occurs at lower temperatures, owing to the decrease in silica solubility with cooling (Posth et al., 2008).

### Stromatolites

Stromatolites are layered carbonate formations that were widespread in marine settings before the rise of animals, although they are restricted to limited settings in the modern ocean due to the existence of grazing and burrowing animals (Garrett, 1970; Bosak et al., 2013; Peters et al., 2017). Although the morphology and texture of Precambrian stromatolites could be similar to those of the modern ocean, the processes for the formation of stromatolites may be different due to the evolution of seawater conditions and the potential of changes in stromatolite-forming microbial communities over time (Bosak et al., 2013). For instance, sulfate reduction is believed to stimulate carbonate precipitation in modern stromatolites, as it can increase the dissolved inorganic carbon (DIC) concentration and thus the saturation index of carbonate. However, the DIC concentration of Precambrian seawater could be much higher than that of modern seawater, thus sulfate reduction would not have had much influence on the DIC concentration at the site of stromatolite formation. Through experiments with sulfate-reducing bacteria under artificial Precambrian seawater, Bosak and Newman (2003) revealed that sulfate-reducing bacteria could promote the formation of carbonate in Precambrian stromatolites by modulating carbonate nucleation rather than DIC concentration.

Cyanobacterial photosynthesis is the driver for microbial activity in modern stromatolites (e.g., Riding et al., 1991). However, the first extensive formation of stromatolites occurred at about 3.43 Ga (Allwood et al., 2006), which could be earlier than the occurrence of oxygenic photosynthesis (e.g., Planavsky et al., 2021). Bosak et al. (2007) experimentally evaluated the hypothesis that anoxygenic photosynthesis could build stromatolites. They found that anoxygenic photosynthetic bacteria could simulate the formation of carbonate crusts for stromatolites and thus may have played an important role in the formation of the earliest stromatolites. However, a later study using different species of anoxygenic phototrophic bacteria suggested the existence of a mechanism that impedes the formation of carbonate by anoxygenic phototrophic bacteria (Bundeleva et al., 2012), resulting in low-efficiency carbonate formation by anoxygenic phototrophic bacteria.

## Future works

Although microbial cultivation experiment can greatly advance our understanding of the interaction between life and environments during the early Earth, there are several pitfalls to using this method. Firstly, as mentioned above, the environmental conditions of the Precambrian were quite different from those of the modern era in multiple aspects, and the artificial Precambrian environments used in cultivation experiments usually only consider a limited number of environmental factors, which cannot fully simulate the actual processes in the Precambrian. For instance, the primary productivity of the Precambrian could be influenced by various environmental forcings such as UV intensity, solar constant, length of daytime, *p*CO_2_, marine *p*H, temperature, nutrient concentrations, and toxic components such as Fe(II). Thus, more constraints on the Precambrian environmental conditions would certainly be beneficial for further experimental simulation, and the consideration of additional environmental factors during the cultivation experiments would increase the reliability of the conclusions.

Secondly, the microbial species of the Precambrian could be different from those of today. Although it has been suggested that the components of well-adapted ecosystems may remain unchanged if there is no change in the physical-biological environment (e.g., Schopf et al., 2015), the environmental settings of the Precambrian were significantly different from those of modern environments and a perfect modern analogue for Precambrian settings is lacking. Thus, it is not known whether the species used in current cultivation experiments did actually exist during the Precambrian. On the other hand, there are a lot of biological mechanisms in the organisms of the early Earth that have now been lost due to evolutionary redundancy under current environmental conditions. Moreover, there are many species with similar functions and yet there are even more species to be discovered in the modern Earth surface, which adds more complexity to the identification of species that are applicable to the Precambrian. For example, a considerable number of photoferrotrophs in both fresh water and seawater are known today, including green sulfur bacteria, purple sulfur bacteria and purple non-sulfur bacteria (Posth et al., 2014 and the references therein). Knowledge of the first group of microorganisms that has developed certain metabolic pathways such as photoferrotrophs and the time of gene transfer between microbial groups would certainly be helpful in the design of simulation experiments. In this case, biomarkers may provide vital information on the existence of specific groups or even species of microbes in rock records. Other methods such as molecular clock and geochemical tracer, could also be helpful in the identification of certain metabolic pathways or groups of microorganisms on early Earth. Meanwhile, the similarities between the environmental settings of modern strains and those of the Precambrian should be evaluated to identify the applicability of certain species to the Precambrian period.

Thirdly, most simulation experiments are based on isolated / culturable species, whereas it is known that most microorganisms are not yet cultivated (Steen et al., 2019). Meanwhile, the effect of a single species may not be applicable to an entire ecosystem. This is due to the existence of competition and mutualistic symbiosis in the ecosystem (e.g., Ozaki et al., 2019; Szeinbaum et al., 2021), which could significantly alter the adaptability of microorganisms to certain environments. Experimental studies on microbial mats usually include the entire microbial ecosystem. However, other studies of simulated Precambrian environments usually involve only one or a few species (e.g., Konhauser et al., 2007; Gauger et al., 2016; Ozaki et al., 2019; Szeinbaum et al., 2021), partially because of the difficulty in choosing the proper combination of microbial strains to mimic the Precambrian ecosystem, as there is no perfect modern analogue. In this case, omics based methods (e.g., metagenomics, metatranscriptomics and metaproteomics) can be used to target multiple functional groups / species, various metabolic pathways and their activities (Ayala-Muñoz et al., 2022; McCain et al., 2022). These culture-independent studies can provide invaluable information for us to understand the potential of microbial life and how microbes thrived in extreme environments analogous to the early Earth, such as hydrothermal vents, the deep subsurface, and serpentinites, which are not easy to sample and are hard or time-consuming (months to years) to simulate in the lab (Jørgensen and Boetius, 2007; Trembath-Reichert et al., 2017). Interestingly, chemoautotrophy is found to be one of the dominant living strategies in many samples from these environments, either through oxidation of CO, reduced sulfur or H_2_ (Fortunato and Huber, 2016; Lecoeuvre et al., 2021; Nobu et al., 2022; Rogers et al., 2022), showing a slow lifestyle and adaption to the limited availability of nutrients and energy. Multi-omics studies not only reveal unknown lineages, metabolic pathways and functions (Hug et al., 2016) but also can help identify new species (Lewis et al., 2020), such as new anaerobic arsenic methylating bacterium (Viacava et al., 2022) or an archaeon at the prokaryote–eukaryote interface (Imachi et al., 2020). Therefore, both culture-dependent and culture-independent methods are needed for future experimental studies of microbial activities in early Earth.

Lastly, the scope of current experimental simulations on the biogeochemical cycles of the early Earth is still limited. Although there are a considerable number of experimental studies simulating the primary productivity of the Archean ocean, experimental simulations on the speed and/or kinetics of other processes in the organic carbon cycle such as oxic weathering, biological pump, and remineralisations in both seawater and sediments, remain limited. Moreover, experimental simulations on the biogeochemical cycle of the other major and trace elements such as N, P and S are also largely lacking. Therefore, further experimental studies on these aspects have the potential to significantly advance our understanding of the biogeochemical cycles of the early Earth and perhaps other extraterrestrial planets that hold life.

## Author contributions

MZ and K-QX discussed the idea. MZ wrote the manuscript and revised it together with YZ, WL, and K-QX. All authors contributed to the article and approved the submitted version.

## Funding

MZ is funded by the 100 Talents program of the Chinese Academy of Sciences (E251520401).This research project has also received funding from the European Research Council (ERC) under the European Union’s Horizon 2020 research and innovation programme (grant agreement no. 725613 MinOrg).

## Conflict of interest

The authors declare that the research was conducted in the absence of any commercial or financial relationships that could be construed as a potential conflict of interest.

## Publisher’s note

All claims expressed in this article are solely those of the authors and do not necessarily represent those of their affiliated organizations, or those of the publisher, the editors and the reviewers. Any product that may be evaluated in this article, or claim that may be made by its manufacturer, is not guaranteed or endorsed by the publisher.
